# Low Serum Alpha-1 Antitrypsin (AAT) in Family Members of Individuals with Autism Correlates with PiMZ Genotype

**DOI:** 10.4137/bmi.s1115

**Published:** 2009-03-18

**Authors:** Anthony J. Russo, Lauren Neville, Christine Wroge

**Affiliations:** Mount Saint Mary’s University

**Keywords:** alpha-1 antitrypsin, autism, gastrointestinal disease

## Abstract

**Aim:**

Deficiency of Alpha-1-antitrypsin (AAT) can be a genetic condition that increases the risk of developing liver, lung and possibly gastrointestinal disease. Since many autistic children also have gastrointestinal disorders, this study was designed to measure serum concentration of AAT and establish AAT genotypes in autistic children, age and gender matched non-autistic siblings, parents and controls.

**Subjects and Methods:**

We used an indirect ELISA with monoclonal IgG to AAT to measure AAT serum concentrations in 71 members from 16 families of individuals with autism and 18 controls (no family history of autism). We used a duplex polymerase chain reaction to detect M, S and Z alleles for alpha-1 antitrypsin expression in 52 members of 12 of the above families.

**Results:**

A significantly high number of autistic family members had lower than normal serum levels of AAT when compared to controls. Autistic children with regressive onset had significantly lower levels of AAT compared to controls, and a significant number of autistic children with low serum AAT also had hyperbilirubinemia, gastrointestinal disease and respiratory problems. We also found that a significantly high number of these individuals had the PiMZ genotype and correspondingly low levels of serum alpha-1 antitrypsin.

**Discussion:**

Knowing that low levels of alpha-1 antitrypsin may be inherited, and that low levels of AAT may be associated with GI disease in autistic children, genotyping autistic children may help identify individuals susceptible to developing digestive problems.

## Introduction

Alpha-1-antitrypsin (AAT) is the most abundant circulating serine protease inhibitor with normal serum concentration of 85–250 mg/dL, but peak concentrations as great as fourfold that of normal may occur during inflammation.^1^–^3^ AAT is a 394 amino acid, 52 kDa glycoprotein synthesized in the liver and secreted into the circulation with a half-life of 4–5 days.^3^

AAT deficiency is a genetic condition that increases the risk of developing a variety of diseases including pulmonary emphysema, cirrhosis of the liver and possibly GI disease. It is caused by mutations in the AAT gene coding^4^–^6^ on the long arm of human chromosome 14 (locus 14q32.1).^7^ Over 100 allelic variants of this gene have been identified and 34 of them have been associated with a quantitative or functional deficiency of circulating AAT.^8^

The protease inhibitor or Pi system has been used to name the various mutations of the AAT gene.^9^ The normal allele is Pi M and the classic severe deficiency is associated with the Pi Z allele. Individuals with the recessive Pi ZZ genotype tend to have circulating levels of AAT, which are 10%–15% of individuals with the Pi MM genotype.^10^ Since the alleles are co-dominant, heterozygotes (PiMZ) have values approximately 35% of the normal concentration. Other genotypes associated with severe deficiency include Pi SZ, Pi Z/Null and Pi Null, as well as an array of much more rare Pi types.^1^–^11^ The population prevalence for the MM, MS, and MZ genotypes among whites are 86, 9, and 3%, respectively.^12^

Alpha-1 antitrypsin (AAT) is mostly secreted by hepatocytes and, to a lesser extent, by lung epithelial cells and phagocytes. It inhibits a variety of serine proteinases, but its preferred target is human neutrophil elastase (HNE), for which it demonstrates the highest affinity.^13^

The major function of AAT in the lungs is to protect the connective tissue from HNE released from triggered neutrophils, as supported by the development of pulmonary emphysema early in life in subjects affected by severe inherited deficiency of AAT.^14^ In the majority of humans, the lungs are defended from HNE attack by normal AAT plasma levels ranging from 85 to 250 mg/dl.^15^ Although AAT is a well-known acute phase reactant, this wide variability in its normal plasma level mostly reflects the marked pleomorphism of the glycoprotein. More than 100 genetic variants of AAT have been identified and these are strictly associated with specific AAT plasma levels in a co-dominantly inherited fashion.^16^–^17^

Alpha-1 antitrypsin production increases markedly after stresses such as surgery, injury, infection, or inflammation and with estrogen administration.^3^ Low values are associated with emphysema, liver disease and possibly gastrointestinal disease.^18^

Toxic chemicals found in cigarette smoke, air pollutants, oxidizing chemicals, and oxidizing agents released by activated neutrophils, all inactivate a critical portion of the alpha-1-antitrypsin molecule.^19^

If autistic children express low levels of alpha-1-antitrypsin.^20^ they may be more susceptible to proteolytic damage caused excess digestive enzymes released from white blood cells during inflammation or infection.^21^

Children with autism frequently have nongastro-intestinal symptoms suggestive of digestive diseased, such as reflux esophagitis.^22^ Infants and children with gastroesophageal reflux disease more frequently have sleep disturbance than the normal population.^23^ Autistic children also have functional gastrointestinal abnormalities. Low activities of disaccharidase enzymes (lactase, maltase, sucrase, palatinase, and glucoamylase) have been found in autistic children.^22^ Abnormal serum liver function tests have been described in children with AD.^24^–^25^ Wakefield et al.^26^ obtained ileocolonic biopsies from 60 consecutive children with developmental disorders, 83% of whom had autism. Fifty-nine had one or more GI symptoms (e.g. abdominal pain, constipation, diarrhea, changing stool consistency [constipation alternating with diarrhea], or bloating. All were well nourished with height and weight within the normal range. Colonic endoscopic findings included segmental swelling, hyperemia, superficial erosions, and nodularity. On histologic examination, mild to moderate ileal lymphoid nodular hyperplasia (LNH) was described in 93% of the developmentally delayed and autistic children examined.

Since some autistic children have GI problems which might be associated with inflammation, we hypothesized that low AAT levels may be associated with these conditions.

In this study, we determined AAT serum concentrations of 71 members from 16 families of individuals with autism, compared these to concentrations of AAT in 18 controls (parents with no family history of autism), and found that a significant number of family members had lower than normal AAT levels (less that 85 mg/dl). Using a duplex polymerase chain reaction, we detected M, S and Z alleles for alpha-1 antitrypsin expression in 52 family members from 12 of the above families. We found a significantly high number of family members (n= 37) with the MZ genotype. A significant number of these heterozygotes also had correspondingly low levels of serum alpha-1 antitrypsin.

Since AAT concentration may be lower than that of the normal population, detection of low serum AAT and establishment of AAT genotype, might be useful tools for assessing prognosis in autism. Also, a better understanding of the incidence of AAT deficiency in autistic families and its potential relationship to gastrointestinal, liver and lung diseases, may lead to better therapy.

## Methods

We used an indirect ELISA and western blotting, as previously described^27^,^28^ to quantitate AAT concentration in serum of autistic and control individuals. We also used a duplex polymerase chain reaction to detect M, S and Z alleles for alpha-1 antitrypsin expression.^29^

### Indirect ELISA to quantitate the concentration of serum AAT

Purified Alpha-1 antitrypsin (Sigma) at concentrations of 1 μg, 0.1μg, 0.01 μg and 0.001 μg per 100 μl of bicarbonate buffer (pH 9.6), and 100 μl serum protein at a dilution of 1:500 (PBS), were fixed to wells of 96 well polystyrene microculture plate (Corning) by incubation overnight at 4 degrees C. Excess AAT/ serum was dumped from wells, and all wells were blocked by washing 3X with 300 μl blocking solution (Superblock, Pierce). One hundred microliters of primary antibody (mouse Mab to AAT (Biomedia), diluted 1:500 with PBS, was added to all wells and plate was incubated for 2 hours at 37 degrees C. All wells were washed 3 times with PBS/tween. One hundred microliters of affinity purified alkaline phosphotase conjugated goat anti mouse IgG (Biorad), diluted 1:5000 with PBS, were added to all wells and incubated for 45 minutes at 37 degrees C. All wells were washed 5 times with PBS/tween. One hundred microliters of AP substrate (Biorad) were added to all wells and the plate was incubated at room temperature until significant color change in positive control (10–15 minutes). Optical density was measured using ELISA Reader (Biorad).

### Western blot to test specificity of monoclonal anti-AAT IgG to purified AAT

#### Protein Preparation

Purified AAT (Sigma), and controls were incubated 1:1 with loading dye (with beta mercaptoethanol) at 95 C for 5 minutes. Protein was run on SDS-Page (18%) (BioRad), at 100 volts, 1 μg/lane.

#### Blot

Proteins were transferred to nitrocellulose at 30 volts, overnight at 4 C. Nitrocellulose was flooded with blocking solution (1% casein/PBS) and rocked gently overnight.

#### Immunoblot

Ten ml of primary antibody (mouse Mab to AAT diluted 1:500 with PBS; Negative control—PBS) was flooded over appropriate nitrocellulose sheets and incubated for 2 hours at room temperature with gentle rocking. Sheets were washed 3 times using PBS/tween. Five minutes for each wash, rocking gently at room temperature. Sheets were flooded with 10 ml of affinity purified goat anti-mouse IgG conjugated with Horse Radish Peroxidase (HrP) (Biorad), diluted 1:5000 with PBS. Sheets were washed 5 times with PBS/tween, five minutes each wash, rocking gently at room temperature. Sheets were flooded with 10 ml of HrP substrate at room temperature until significant color change.

### Genotyping of autistic and nonautistic family members using pcr and restriction enzyme analysis

Polymerase chain reactions was performed with standard buffer (0.1 μ Tris-HCl, pH = 8.3, 0.5 μ KCl and 10 mμ MgCl2). in a total volume of 100 μl containing: 250 ng of genomic DNA, 0.25 pM each of the oligonucleotide primers, 0.2 mμ of dNTPs and 2.5 U of Taq polymerase. Primers used 11detectingtheSmutationare:5′-TGAGGGGAAAC-TACAGCACCTCG-3′ and 5′AGGTGT-GGGCAGCTTCTTGGTCA-3′; primers detecting the Z mutation are: 5′-ATAAGGCTGTGCTGAC-CATCGTC-3′ and 5′-TTGGGTGGGATTCAC-CACTTTTC-3′. Temperature cycling conditions were: initial 10 min denaturation at 94 °C, 30 cycles of 2 min at 94 °C, annealing for 2 min at 55 °C, extension for 3 min at 72 °C, and final extension for 10 min at 72 °C. Twenty microlitres of amplification product were digested for 3 h at 65 °C in a 50 μl volume containing 20 U of TaqI restriction enzyme, in the appropriate buffer. The digested DNA was electrophoresed in a 3% agarose minigel for 3 h at 90 volts, stained with ethidium bromide and visualized under 254 nμ uv trans-illumination.

#### Patient group

Seventy-one family members from 16 families of individuals with autism, obtained from the Autism Genetic Resource Exchange (AGRE, see below), and 18 controls (parents with no family history of autism), obtained from National Disease Research Interchange (NDRI, see below) were tested for their serum concentration of alpha-1 antitrypsin and AAT genotype.

## Results

### AAT concentration

**Figure 1:**
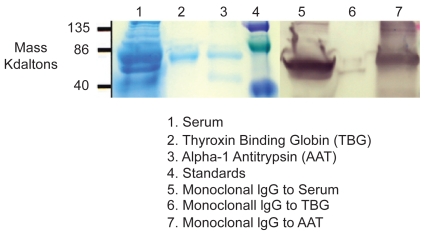


**Figure 2:**
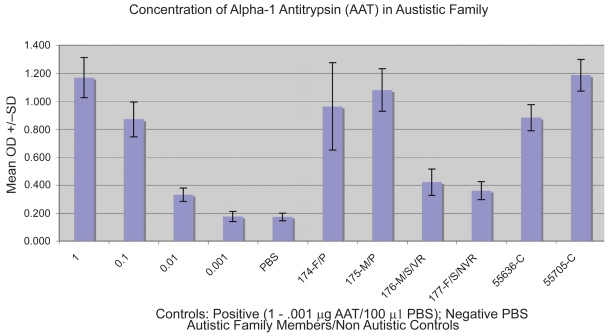


Using an indirect ELISA, serum from individuals was tested using anti-AAT monoclonal IgG and compared to varying concentrations (1 μg–0.001 μg/) of purified AAT and negative control (PBS). Specificity of monoclonal anti-AAT IgG was established using western blotting (Fig. 1). Results of a typical ELISA are shown on Figure 2.

**Figure 3:**
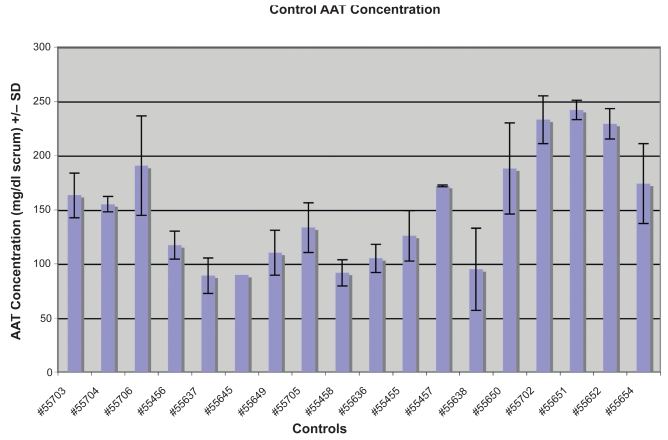


Forty-five of the 71 individuals from the 16 families had AAT concentrations less than 85 mg/dL serum, making them low producing individuals (Table 1). Whereas none of the 18 non-autistic controls had levels of AAT less than 85 mg/dL (Fig. 3) (p < 0.05).

**Figure 4:**
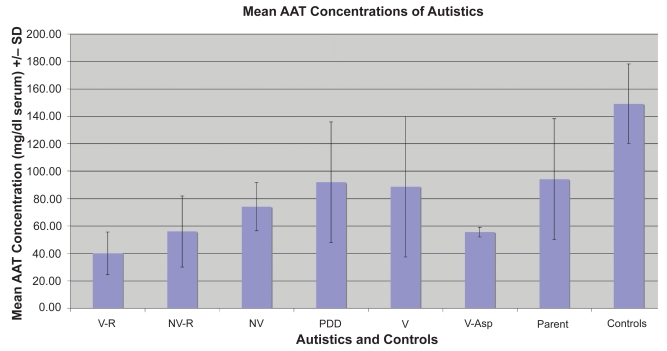


Comparing AAT levels in parents and autistic children with a variety of demographics (Fig. 4 and Table 2), a significantly high number of autistic family members (45/72) had lower than normal levels of AAT when compared to controls (0/18) (p < 0.01). Of the types of autistics tested, we found that non verbal (11) and non verbal regressive (8), autistic siblings had significantly lower levels of AAT compared to controls (13) (p < 0.05).

Using medical history reports (gathered by AGRE), we analyzed the phenotypic characterization of the probands of family members tested in this study (Table 2). Overall, twenty-seven autistic children were tested for AAT concentration. Questions associated with hyperbilirubinemia were not answered for five of these children. Of the 22 remaining, 8 had hyperbilirubinemia at birth (36%). This is significantly higher than the expected 6 percent of normal children who have the disorder. Six of the remaining 8 with hyperbilirubinemia had low levels of AAT (less than 85 mg/dL) (p < 0.05). Two of the 8 with hyperbilirubinemia required phototherapy.

Three of the 23 affected individuals who answered the related questions had respiratory distress syndrome (RDS) (p < 0.05). Two of these three were born after short gestation (29 weeks). All three required oxygen supplimentation. None of these individuals had hyperbilirubinemia. Two of these three had deficient levels of AAT.

Six other affected siblings (not including the three with RDS) reported respiratory problems later in life. Five of these 6 had deficient levels of AAT. Although none of the individuals had emphysema, two reported multiple respiratory problems—tracheomalacia and sleep apnea, stridor and laryngomalacia. One reported reactive airway disease. One reported multiple incidences of pneumonia and the other two did not report a specific disorder.

Seven of 22 affected siblings reported gastrointestinal problems. One of these did not answer the question. Five of the remaining six had deficient levels of AAT (p < 0.05). Three of the six reported chronic diarrhea, one reported constipation, one colic and the other, projectile vomiting.

### Genotyping

If we predicted the genotype of all 71 members of the 16 autistic families based on their AAT serum concentrations (Table 1), most (43/71) would be heterozygous (30–85 mg/dL). This is significantly higher than the expected 3% in the general population. Two pairs of identical twins and three pairs of fraternal twins had very similar AAT concentrations and the same predicted genotypes. In five of six families, when parents had deficient levels of serum AAT (less than 85 mg/dL), all their children were deficient. When both parents were heterozygous, all their children were heterozygous or normal. These findings suggest an inheritance pattern of this trait in these autistic families.

**Figure 5:**
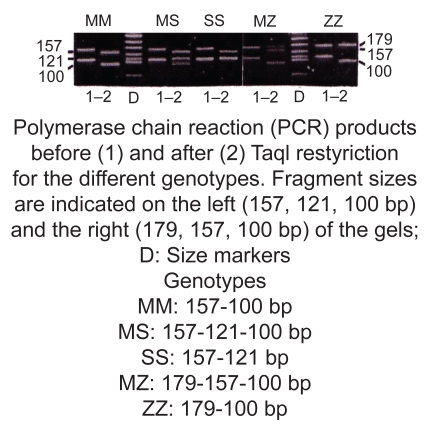


**Figure 6:**
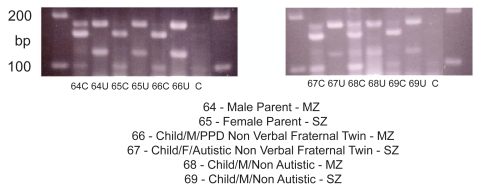


Using a PCR/Restriction Digest we genotyped 52 members from 12 of the 15 families who had been measured for serum AAT concentration (above) (DNA was not available for three of the families). Expected product demonstrating specific MM, MS, SS, MZ and ZZ genotypes for AAT expression is shown in Figure 5. The results of a typical assay (family 64–69) are shown in Figure 6. In this assay, the gel photo shows uncut and cut product for each family member and corresponding genotype.

A significantly high number of family members (37) had the MZ genotype. A significant number of these heterozygotes (35 of 37) had correspondingly low levels of serum alpha-1 antitrypsin (less than 100 mg/dL) (Table 1).

## Discussion

Approximately 75 allelic variants have been described in the AAT gene locus resulting in a very complex genetic classification based upon the phenotypic features of the circulating AAT protein. The most common variant, PI M, is present in approximately 95% of the Caucasian U.S. population and is regarded as the normal variant associated with normal serum levels of functional AAT. The predominant deficiency alleles, such as PI Z and PI S, may result in decreased levels of circulating AAT but with completely normal functioning proteins. The MM phenotype is therefore designated as manifesting 100% concentration of circulating AAT (85–250 mg/dL). The heterozygous combinations MZ and MS yield 35% and the ZZ allele 10%–25% of this normal MM value. Approximately 95% of all AAT deficiency states leading to clinical manifestations are made up of PI ZZ homozygotes. Certain alleles, such as the S allele, either in the homozygous state or associated with the M allele, do not appear to be associated with the abnormally polymerized molecules within the endoplasmic reticulum and have not been incriminated in the development of liver disease unless combined with the Z allele.^14^,^30^

The association of AAT deficiency and liver disease in children was first described in 1969.^31^ Many subsequent clinical studies 32,18 have observed that liver disease occurrence in AAT deficiency is bimodal, affecting children in neonatal life or early infancy and, less commonly, adults in late middle life. However, only 10% of deficient newborns develop persistent cholestasis during the first year of life and many of these infants appear to undergo a spontaneous remission, with only about 3% of the originally diagnosed neonates progress to fibrosis or cirrhosis during childhood and teenage years. Nevertheless, careful surveillance revealed that many of these have persistently abnormal liver enzymes.^30^,^31^

Cederlund and Gillberg (2004),^33^ reported neonatal hyperbilirubinemia in 22 of 100 boys with Asperger syndrome. Bilirubin gets into neurons only if the blood-brain barrier is disrupted by some other factor.^34^,^35^ Some of the newborns with AAT deficiency, who develop jaundice, may suffer anoxia or sepsis in the perinatal period, and may therefore be at risk for bilirubin crossing the blood-brain barrier. Our data shows a significant relationship between low AAT levels and neonatal hyperbilirubinemia in autistic children.

Asperger first recorded the link between celiac disease and behavioral psychoses^36^ Walker-Smith and colleagues detected low concentrations of alpha-1 antitrypsin in a small group of children with typical autism,^20^ and D’Eufemia and colleagues identified abnormal intestinal permeability, a feature of small intestinal enteropathy, in 43% of a group of autistic children with no gastrointestinal symptoms, but not in matched controls.^37^ Some have also found a relationship between chronic enterocolitis and regressive developmental disorder.^38^ These studies and evidence of anemia and immune deficiency in some autistic children, suggest an association between intestinal dysfunction and autism spectrum disorders (ASD). However, increased incidence of gastrointestinal problems in autistic children is still under study because the exact relationship between GI symptoms and ASD is unclear.^39^ Our data, however, suggests that a high number of autistic children with low levels of AAT may also have gastrointestinal problems, especially diarrhea.

In inflammatory conditions, AAT levels are normally elevated. In this study, however, the results demonstrate low alpha-1-antitrypsin serum levels, which are probably due to genetic factors and not excess protein loss in stool. This might contradict the theory that autistic patients have inflammatory bowel disease because if there was chronic inflammation due to enterocolitis, the alpha-1-antitrypsin level should be elevated. However, there is evidence that increased inflammation is associated with AAT deficiency, such as GI inflammation in patients with ulcerative colitis, associated with the PiZ carriers,^40^ and neutrophil associated inflammation seen in the lungs of AAT deficient patients.^41^ Also, PiMZ subjects without airflow obstruction may have an IL-8 related neutrophilic inflammation in the pulmonary airways 42 and they are more likely to develop both liver (cirrhosis or fibrosis) and lung disease (emphysema).^43^

In this preliminary study, a significant number of autistic family members have low serum levels of AAT, which is, at least in part, caused by the PiMZ genotype. Low serum AAT in autistic children supports the findings of Walker-Smith and colleagues, and strongly suggests an association between autism and AAT deficiency.

A high number of autistic children with low levels of AAT in our study, also had neonatal hyperbilirubinemia (8/22), respiratory problems (9/23) and digestive disorders (7/22). Since AAT deficiency may be associated with these problems, this also supports our hypothesis of an association between AAT deficiency and autism.

Our study also suggests an association between autistic children with regressive disease and AAT deficiency. This suggests that, besides genetic predisposition, environmental factors may be influencing levels of serum AAT in these individuals.

Our observation, however, that some non-autistic siblings inherit AAT deficiency, and parents of autistic children who also have low levels of serum AAT, suggests that AAT deficiency alone is not a causative agent for ASD, but may make a subset of autistics susceptible to inflammatory disease.

Knowing that low levels of alpha-1 antitrypsin is inherited, and that low levels of AAT may be associated with GI disease, genotyping autistic children may help identify those autistics susceptible to developing digestive problems. And a better understanding of the incidence of AAT deficiency in autistic families and its potential relationship to liver, lung and bowel diseases, may lead to better therapeutic strategies.

## Figures and Tables

**Table 1:** 

	Diagnosis	Sex	Twins	Relationship	AAT mg/dL	Genotype
Family 1		F		Parent	95	
		M		Parent	54	
	Asp	M		Sibling	58	
	AR	M	IT	Sibling	49	
	A	M	IT	Sibling	47	
Family 2		F		Parent	45	
		M		Parent		
	AR	M		Sibling	51	
	AR	M		Sibling	54	
	NA	F		Sibling	44	
Family 3		F		Parent	22	MZ
		M		Parent	24	MZ
	AR	F	FT	Sibling	38	MZ/Null
	PDD	M	FT	Sibling	46	MZ
Family 4		F		Parent	54	
		M		Parent	65	
	NA	M		Sibling	61	
	A	F		Sibling	73	
	A	F		Sibling	45	
Family 5	PDD	M		Sibling	176	
	AR	M		Sibling	100	
		M		Parent	72	
		M		Parent	74	
	PDD	F		Sibling	76	
Family 6		M		Parent	126	MZ
	A	M		Sibling	123	MM
	PDD	F		Sibling	86	MZ
	A	M		Sibling	78	MZ
		F		Parent	141	MM
Family 7	A	M		Sibling	93	
	A	M		Sibling	133	MM
		F		Parent	88	MZ
		M		Parent	117	MM
Family 8		F		Parent	124	MZ
		M		Parent	104	SZ
	PDD	M	FT	Sibling	78	MZ
	A	F	FT	Sibling	70	SZ
	NA	M		Sibling	146	MZ
	NA	M		Sibling	68	SZ
Family 9	NA	F		Parent	146	MM
	NA	M		Parent	214	MM
	PDD	M		Sibling	89	MM
	NA	F		Sibling	197	MM
	AR	M		Sibling		
Family 10	NA	F		Parent	97	SZ
	NA	M		Parent	94	MZ
	A	F		Sibling	55	MZ
	A	M	IT	Sibling	129	MS
	A	M	IT	Sibling	100	MS
Family 11	NA	F		Parent	46	MZ
	NA	M		Parent	51	MZ
	AR	F		Sibling	29	MZ
	AR	M		Sibling	18	MZ
Family 12	A	F		Sibling	84	MZ
	NA	F		Parent	84	MZ/ZZ
	Asp	M		Sibling	53	MZ
	NA	M		Parent	88	MZ
Family 13	NA	F		Parent	54	MZ
	NA	M		Parent	23	MZ/ZZ
	A	M		Sibling	15	MZ
	A	M		Sibling	35	MZ
Family 14	NA	F		Parent	82	MZ/SZ
	NA	M		Parent	92	MZ
	AR	M		Sibling	36	MZ
	AR	F		Sibling	31	MZ
Family 15	NA	F		Parent	50	MZ
	NA	M		Parent		
	NA	F		Sibling	49	MZ
AR	M	FT		Sibling	51	MZ
	A	M	FT	Sibling	51	MZ
Family 16	NA	F		Parent	171	MM
	NA	M		Parent	93	MZ
	AR	M		Sibling	51	MZ
	A	M		Sibling	57	MZ

**Table 2:** 

	Diagnosis	Sex	Twins	Relationship	AAT	Genotype	HB	HB RX	RD	O2	GI	GI T
1					mg/dL							
2	AR	M	IT	Sibling	49		0	−1	0	0	0	−1
3	A	M	IT	Sibling	47		0	−1	0	0	0	−1
4	AR	F	FT	Sibling	38	MZ/Null	1	−1	0	−1	0	−1
5	PDD	M	FT	Sibling	46	MZ	1	−1	0	0	−1	−1
6	PDD	M		Sibling	176		0	−1	0	0	0	−1
7	AR	M		Sibling	100		1	−1	0	0	1	5,6
8	PDD	F		Sibling	76		0	−1	0	0	0	−1
9	A	M		Sibling	123	MM	0	−1	0	−1	0	−1
10	PDD	F		Sibling	86	MZ	−1	−1	−1	−1	0	−1
11	A	M		Sibling	78	MZ	1	1	0	0	0	−−1
12	A	M		Sibling	93	MM/MZ	0	−1	0	0	0	−1
13	A	M		Sibling	133	MM	1	1	0	0	0	−1
14	PDD	M		Sibling	89	MM	0	−1	0	0	0	−1
15	A	F		Sibling	55	MZ	0	−1	1	1	−1	−1
16	A	M	IT	Sibling	129	MS	0	−1	1	1	−1	−1
17	A	M	IT	Sibling	100	MS	−1	−1	−1	−1	−1	−1
18	AR	M		Sibling			1	PT	0	0	1	5
19	AR	F		Sibling	29	MZ	−1	−1	−1	−1	1	887
20	AR	M		Sibling	18	MZ	−1	−1	−1	−1	1	6
21	A	F		Sibling	84	MZ	1	PT	0	0	1	5
22	Asp	M		Sibling	53	MZ	0	−1	0	0	1	6
23	A	M		Sibling	15	MZ	1	1	0	0	0	−1
24	A	M		Sibling	35	MZ	0	−1	0	0	0	−1
25	AR	M	FT	Sibling	51	MZ	−1	−1	1	1	−1	−1
25	A	M	FT	Sibling	51	MZ	1	−1	0	0	0	−1
27	AR	M		Sibling	51	MZ	0	−1	0	0	1	5
28	A	M		Sibling	57	MZ	0	−1	0	0	0	−1

Key												
Hyperbilirubinemia and Respiratory History												
Select Value				Stored Value			Answer Text					
No				0			No					
Yes				1			Yes					
N/A				9			N/A					
Blank				−1			Indicates that no response was entered on the collection form/instrument for this question					
HB—Hyperbilirubinemia												
HB-RX—Hyperbilirubinemia Therapy												
PT—Phototherapy												
RD—Respiratory Distress												
O2—Oxygen Suppliment												
GI History												
Gastroesophageal Reflux (Ger)				1						
Peptic Ulcer Disease (Pud)				2						
Irritable Bowel Syndrome (Ibs)				3						
Inflammatory Bowel Dis. (Ibd)				4						
Chronic Diarrhea				5						
Constipation				6						
Other				887						
Unknown				888						
Multiple				889						
GI—Gastrointestinal Problem(s)												
GI T—Gastrointestinal Problem Type												
GI D—Gastrointestinal Problem Description												
